# Physiological mechanisms involved in maintaining the corpus luteum during the first two months of pregnancy

**DOI:** 10.21451/1984-3143-AR2018-0045

**Published:** 2018-08-03

**Authors:** Milo C. Wiltbank, Megan A. Mezera, Mateus Z. Toledo, Jessica N. Drum, Giovanni M. Baez, Alvaro García-Guerra, Roberto Sartori

**Affiliations:** 1 Department of Dairy Science, University of Wisconsin-Madison, Madison, Wisconsin, 53706 USA; 2 Endocrinology-Reproductive Physiology Program, University of Wisconsin-Madison, Madison, Wisconsin, 53706 USA; 3 Department of Animal Science, University of São Paulo, Piracicaba, SP, 13418-900, Brazil; 4 Department of Agricultural and Animal Sciences, Universidad Francisco de Paula Santander, Cucuta, Colombia; 5 Department of Animal Sciences, The Ohio State University, Columbus, Ohio, 43210 USA

**Keywords:** corpus luteum, interferon-tau, pregnancy.

## Abstract

Maintenance of the corpus luteum (CL) during pregnancy is essential for continuing the elevated circulating progesterone (P4) that is required to maintain pregnancy. The mechanisms that protect the CL during early pregnancy when the non-pregnant animal would typically undergo CL regression have been extensively investigated. It is clear uterine prostaglandin F2α (PGF) causes regression of the CL in non-pregnant ruminants and that maintenance of the CL during early pregnancy is dependent upon secretion of interferon-tau (IFNT) from the elongating embryo. A number of specific mechanisms appear to be activated by IFNT. Most studies indicate that there is an inhibition of oxytocin-induced secretion of uterine PGF. There is also evidence for increased resistance to PGF action, perhaps due to secretion of PGE2 and PGE1 or direct endocrine actions of circulating IFNT. These mechanisms occur concurrently and each may help to maintain the CL during the first month of pregnancy. However, during the second month of pregnancy, IFNT is no longer secreted by the embryo. Attachment of the embryo to the uterus and subsequent placentome development have been linked to silencing of expression from the IFNT gene. In addition, there is some evidence that oxytocin responsiveness of the uterus returns during the second month of pregnancy leading to substantial basal secretion of PGF and perhaps PGF pulses. There is also no evidence that the CL during the second month of pregnancy is resistant to the actions of PGF as observed during the first month. Thus, this manuscript attempts to compare the mechanisms that maintain the CL during the first and second months of pregnancy in ruminants and provides a new, speculative, physiological model for maintenance of the CL during month two of pregnancy that is distinct from the previously-described mechanisms that maintain the CL during the first month of pregnancy.

## Introduction

**Figure 1:**
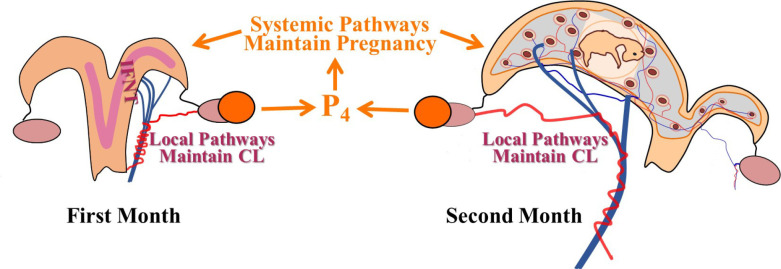
Simplified diagram illustrating that progesterone (P4) maintains the pregnancy through systemic pathways while the pregnancy, either during the first month or second month of pregnancy maintains the CL through local pathways within the utero-ovarian system.

In ruminants, progesterone (P4) is produced by the corpus luteum (CL) and is essential for the original establishment and subsequent maintenance of pregnancy throughout gestation (Wiltbank *et al.,* 2014). Likewise, the embryo/placentomes is essential for maintaining the CL after the first two weeks of pregnancy (Bazer *et al.,* 1997; Spencer *et al.*, 2007; Giordano *et al.*, 2012). Thus, the CL and the pregnancy have a co-dependent relationship that involves both long distance (systemic) and adjacent (local pathways) interactions (Fig. 1). It is well-established that production of IFNT by the elongating embryo maintains the CL during the classical maternal recognition of pregnancy period in the first month of pregnancy (Bazer *et al.,* 1997; Spencer *et al.*, 2007; Wiltbank *et al.*, 2016a). During the second month of pregnancy and beyond, the CL is maintained by mechanisms that remain to be elucidated (Wiltbank *et al.,* 2016b).

Maintenance of pregnancy or, conversely, pregnancy loss can be viewed from two general perspectives (Giordano *et al.*, 2012). First, the embryo/pregnancy may be defective and therefore it is lost, which may be positive for reproductive efficiency because, it is unlikely that a viable offspring would be produced from that pregnancy. The sooner the pregnancy can be recognized as non-viable and discarded, the sooner a new, potentially viable, pregnancy can be initiated. Alternatively, pregnancy loss could occur because the CL inappropriately regresses, resulting in loss of a viable pregnancy, with a subsequent delay in the establishment of a new viable pregnancy, and therefore a reduction in reproductive efficiency. Previous studies have quantified the amount and timing of pregnancy loss in lactating dairy cows, beef cattle, and recipients of *in vitro*-produced (IVP) or *in vivo*-derived (IVD) embryos (Santos *et al.*, 2004; Diskin *et al.*, 2016; Wiltbank *et al.*, 2016a). However, no studies have clearly differentiated if a defective embryo or inappropriate CL regression underlies pregnancy loss in month one or two of pregnancy.

Pregnancy loss during the second month of gestation is substantial (Diskin and Morris, 2008; Santos *et al.*, 2009; Diskin *et al.*, 2016; Wiltbank *et al.*, 2016a). To determine the current magnitude of the problem, we analyzed recent data or recently published studies that evaluated pregnancy loss in lactating dairy cattle (Table 1) or in embryo transfer recipients (Table 2).

**Table 1:** Studies that evaluated pregnancy/AI (P/AI) and pregnancy loss between the first (27-34 days after AI) and second pregnancy diagnosis (53-74 days after AI) in primiparous and multiparous cows or overall pregnancy loss in dairy cows from 2005 to 2017.

Pregnancy diagnosis, day				P/AI at 1st diagnosis, % (n breedings)		Pregnancy loss % (n pregnancies)		Overall pregnancy loss % (n/n)
Reference^1^	Year	1st	2nd	Primiparous	Multiparous	Primiparous	Multiparous	
Baez	2016	32	67	52.2 (289)	42.8 (327)	4.6 (153)	8.6 (140)	6.5 (19/293)
Bartolome	2005	30	55	-	-	-	-	8.5 (10/118)
Bartolome	2009	30	55	29.1 (302)	39.1 (416)	11.4 (88)	10.4 (163)	10.8 (27/251)
Bartolome	2005	27	55	-	-	-	-	18.7 (67/358)
Bilby	2013	32	60	-	-	-	-	10.5 (60/569)
Bisinotto	2015	32	60	-	-	-	-	9.7 (68/699)
Bisinotto	2015	32	60	-	-	-	-	8.0 (56/704)
Bruno	2009	31	66		35.4 (717)		20.1 (254)	20.1 (51/254)
Carvalho	2014	32	70	51.0 (224)	46.0 (377)	5.2 (115)	7.0 (172)	6.3 (18/287)
Carvalho	2015	32	67	57.5 (240)	53.3 (553)	9.4 (138)	9.7 (289)	9.6 (41/427)
Dirandeh	2015	32	60	-	41.0 (900)	-	15.4 (369)	15.4 (57/369)
Dirandeh	2014	32	60	-	28.1 (459)	-	6.2 (129)	6.2 (8/129)
Dirandeh	2015	32	60	-	17.7 (1374)	-	7.0 (243)	7.0 (17/243)
Giordano	2013	29	74	53.8 (519)	40.7 (565)	-	-	15.5 (79/509)
Giordano	2012	29	74	-	-	-	-	15.4 (101/654)
Giordano	2012	29-32	53	-	-	-	-	7.8 (58/737)
Giordano	2015	31	67	-	-	-	-	11.3 (37/326)
Hernandez	2012	28-32	56-60	-	-	12.7 (173)	19.5 (339)	17.0 (88/512)
Karakaya	2014	31	62	47.6 (126)	28.4 (176)	13.3 (60)	8.0 (50)	10.9 (12/110)
Lima	2009	28-32	56	-	-	-	-	12.7 (108/849)
Lima	2012	32	60	-	-	-	-	16.8 (68/405)
Lopes Jr	2013	32	64	-	-	-	-	5.1 (20/394)
Martinez	2016	32	60	45.3 (168)	43.1 (274)	8.3 (76)	11.6 (118)	10.3 (20/194)
Melo	2016	32	60	-	-	-	-	14.3 (52/363)
Monteiro	2015	32	60	35.9 (298)	28.6 (370)	18.7 (107)	17.9 (206)	18.3 (39/213)
Monteiro	2014	34	62	-	-	-	-	8.0 (39/487)
Pereira	2014	32	60	-	-	-	-	11.1 (37/333)
Pereira	2013	32	60	-	-	-	-	15.7 (40/254)
Pereira	2013	30	71	-	-	-	-	11.8 (40/338)
Pereira				-	-	-	-	10.7 (104/968)
Pontes	2015	31	62	-	-	-	-	16.3 (46/283)
Ribeiro	2012	30	65	-	-	-	-	9.5 (96/1016)
Santos	2017	33	63	54.6 (196)	38.4 (318)	6.5 (107)	9.0 (122)	8.3 (19/229)
Toledo	2017	28	61	65.0 (126)	66.9 (157)	13.8 (80)	13.0 (100)	13.3 (24/180)
Vieira-Neto	2017	32	60	40.3 (3242)	36.8 (4853)	9.1 (1306)	14.4 (1784)	12.2 (376/3090)
Overall^2^				43.0^x^	36.1^y^	9.5^w^	13.4^z^	11.7
				(2509/5830)	(4280/11836)	(229/2403)	(586/4378)	(2002/17145)

1 Only first author listed.

2 Overall P/AI and pregnancy loss of primiparous vs multiparous cows differ (P < 0.01).

**Table 2:** Recent studies (since 2009) that evaluated pregnancy/embryo transfer (P/ET) and pregnancy loss between the first (28-38 days of pregnancy) and second pregnancy diagnosis (50-60 days of pregnancy) in various breeds and categories of cattle.

Pregnancy diagnosis, day							
Reference^1^	Year	1st	2nd	Breed	Category	P/ET at 1st diagnosis % (n/n)	Pregnancy loss % (n/n)
*In vitro* produced (IVP)							
Breukelman	2012	36	50	Holstein	Dry cows	52.4 (76/145)	14.5 (11/76)
Garcia-Guerra	2017	32	60	Holstein	Heifers	43.4 (6539/15052)	19.8 (1293/6539)
Gatea	2018	30	60	Girolando	Lactating cows	NR^2^	16 (21/131)
Munhoz	2014	30	60	Gyr	Heifers/Lactating cows	52.6 (950/1807)	17.9 (170/950)
Pereira	2017	32	60	Holstein/Girolando	Lactating cows	31.0 (100/323)	19.0 (19/100)
Pereira	2016	32	60	NR	Lactating cows	41.8 (838/2003)	19.5 (163/838)
Pohler	2016	31	59	Girolando	Lactating cows	NR	16.5 (47/285)
Pontes	2009	30	60	Nelore	Heifers	37.4 (341/910)	10.5 (36/341)
Pontes	2011	30	60	Nelore x Simmental	Heifers	36.6 (1974/5398)	9.4 (186/1974)
Randi	2015	30	60	NR	NR	43.5 (2065/4749)	6.1 (127/2065)
Rasmussen	2013	32-35	60	Holstein	Lactating cows	25.9 (57/220)	26.8 (15/56)
Total						43.5 (10966/25209)	15.6 (2088/13355)
*In vivo* derived (IVD)							
Baruselli	2011	30	60	Holstein	Lactating cows	39.3 (2109/5364)	20.5 (432/2109)
Breukelman	2012	36	50	Holstein	Dry cows	53.9 (62/115)	4.8 (3/62)
Pereira	2013	28	60	Holstein	Lactating cows	44.4 (216/487)	17.6 (38/216)
Pontes	2009	30	60	Nelore	Heifers	45.6 (132/289)	9.0 (12/132)
Rodrigues	2010	30	60	Holstein	Lactating cows^*^	43.4 (159/366)	7.5 (12/159)
Vasconcelos	2011	28	60	Holstein	Lactating cows	45.4 (298/657)	16.4 (49/298)
Wallace	2011	31-33	55/59	Beef mixed	Mature cows	55.6 (198/356)	3.5 (7/198)
Total IVD						41.6 (3174/7634)	17.0 (541/3174)

* Repeat breeders.

1 Only first author listed.

2 NR = not reported.

In lactating dairy cows, analysis of studies from 2005 to 2017 demonstrated an average pregnancy loss of 11.7% (2,002 losses of 17,145 confirmed pregnancies). In 10 of these studies, there was a direct comparison of pregnancy per AI (P/AI) and pregnancy loss in primiparous and multiparous cows. Primiparous cows had ~20% greater P/AI than multiparous at first pregnancy diagnosis (43.0 *vs*. 36.1 = 6.9% absolute difference; 6.9/36.1 = 19.1% relative difference). However, the difference was even greater at second pregnancy diagnosis (primiparous = 38.9% *vs*. multiparous 31.3%) due to ~40% greater pregnancy loss in multiparous than primiparous cows (3.9/9.5 = 41.1%).

Pregnancy loss is also a substantial problem in embryo transfer recipients (Table 2). Based on data from over 25,000 embryo transfers, researchers that transferred IVP embryos, had over 40% P/ET at the first pregnancy diagnosis with subsequent loss of 15.6% of confirmed pregnancies during the second month. Published studies with IVD embryos also had over 40% P/ET at first pregnancy diagnosis and 17% pregnancy loss during second month of pregnancy. These studies were not direct comparisons of IVP *vs*. IVD embryos but are shown to illustrate that pregnancy loss is a substantial problem in either IVP or IVD embryo transfer. Clones have even more problems in this period, with ~50% of confirmed pregnancies lost during the second month (Sala *et al.*, 2018; University of Wisconsin-Madison and ST Technology; unpublished; 51.9%; 82/158). Thus, the second month of pregnancy is a pivotal period for pregnancy loss and could be a substantial area of opportunity, particularly if the problem is due to inappropriate regression of the CL during this period.

In cattle, if pregnancy does not occur, regression of the CL is initiated between day 16 and 25 after ovulation due to secretion of pulses of PGF from the uterus in response to circulating oxytocin pulses (Ginther *et al.*, 2012; Spencer and Hansen, 2015; Wiltbank *et al.*, 2016b). In pregnant cattle, during this same time period (i.e. during the first month of pregnancy) the CL does not undergo regression due to the actions of IFNT which is secreted by the elongating embryo near the time when luteolysis would be expected to occur (Thatcher *et al.*, 1989; *Plante et al.,* 1991; Meyer *et al.*, 1995; Spencer and Bazer, 1996).

However, the mechanisms that maintain the CL during the second month of pregnancy are incompletely defined. This is despite the potential practical value of this research since it could lead to a rational method for reducing the substantial and economically-costly effects of pregnancy loss during this pivotal period. Unraveling these mechanisms could also provide intriguing fundamental biological information that could be of value in other species, including humans. This review will explore four key principles related to maintenance of CL during the first month of pregnancy including: 1) involvement of local pathways, 2) role of IFNT, 3) patterns of uterine PGF secretion, and 4) resistance to PGF action. We will then review these same potential mechanisms in maintaining CL during month two of pregnancy and perhaps later pregnancy. Our purpose in writing this review is to stimulate research on maintenance of the CL after the original maternal recognition of pregnancy period.

## Local utero-ovarian pathways in protection of the CL during pregnancy

### First month of pregnancy

The local pathways for maintenance of the CL during pregnancy are interconnected with the local pathways that produce regression of the CL during the normal estrous cycle. Early studies demonstrated that the non-pregnant uterus was the initiator of the luteolytic process in the guinea pig (Loeb, 1927). Studies in ruminants also found that removal of the uterus greatly prolonged, perhaps indefinitely, the lifespan of the CL, demonstrating the pivotal role of the uterus in luteolysis (Wiltbank and Casida, 1956). In addition, ipsilateral (uterine horn on same side as CL) hysterectomy invariably prolonged the lifespan of the CL, while contralateral (opposite side from CL) hysterectomy consistently failed to affect CL lifespan (Inskeep and Butcher, 1966). This clearly demonstrated that local pathways between uterus and ovary were involved in initiating CL regression. The ovarian artery in ruminants is extremely convoluted and in close apposition to the uterine vein, thus allowing transfer of the uterine luteolysin, PGF, to the ovarian artery (Ginther and Delcampo, 1974; Mapletoft *et al.*, 1976a). Elegant vascular anastomoses studies were done by exchanging the uterine veins after unilateral hysterectomy. These studies demonstrated that the intact uterine horn secreted a luteolysin into the uterine vein that subsequently diffused, through a local pathway, to the ovarian artery and caused luteolysis (*Mapletoft et al., 1976a* ). Nevertheless, well-designed physiological studies done in sheep during the late luteal phase and following infusion of a low dose of oxytocin indicate that there are multiple pathways by which PGF secreted from the uterus can reach the CL (Bonnin *et al.*, 1999). Determinations of PGF flow rates from the uterus, into the lungs and subsequently arriving at the ovary were done by catheterizing the uterine vein, pulmonary artery, femoral artery, and ovarian artery near the ovarian hilus (distal ovarian artery). Treatment with oxytocin increased PGF concentrations in uterine vein (3,811 pg/ml), pulmonary artery (before lungs; 224 pg/ml), femoral artery (after lungs; 18 pg/ml), and distal ovarian artery (42 pg/ml). Only 0.05% of uterine- secreted PGF reached the ovary (1/2000 of PGF released) with one-third of the PGF arriving rapidly by a systemic route (PGF not metabolized in lungs) and two- thirds arriving by slower routes involving local diffusion (Bonnin *et al.*, 1999). Thus, the majority of PGF involved in regressing the CL arrives through local mechanisms, although some PGF may arrive from systemic circulation during a PGF pulse.

There are now multiple types of evidence that uterine-derived PGF is the definitive initiator of CL regression in ruminants and that PGF crosses from uterine vein to ovarian artery through local pathways that initiate the luteolytic process (Knickerbocker *et al.*, 1988; Bonnin *et al.,* 1999; Wiltbank *et al.*, 2016b). Studies using [^3^H]-PGF demonstrated that during PGF pulse peak, sufficient PGF is transported from uterine vein to ovarian artery to initiate the luteolytic process (Lamond *et al.*, 1973; McCracken *et al.*, 1981). Indeed, intrauterine treatment with pulses of PGF, that mimic the natural PGF pulses, can induce CL regression that resembles natural luteolysis (Schramm *et al.*, 1983; Ginther *et al.*, 2009; Atli *et al.*, 2012; Ochoa *et al.*, 2018). Transport of PGF between the utero-ovarian vein and the ovarian artery involves a specific PG transporter, termed SLCO2A1 or OATP2A1 (*Kanai et al., 1995;*Schuster, 1998, 2002) that has 12- transmembrane domains and provides efficient and specific transport of PGF and PGE between these vessels (Lee *et al.*, 2010, 2013; McCracken *et al.*, 2011). The remainder of PGF continues to be transported by the uterine vein into the systemic circulation, the heart, with eventual passage through the lungs in which ~88% of PGF will be metabolized (Bonnin *et al.*, 1999), primarily to the inactive PGF metabolite (PGFM) by the enzyme prostaglandin dehydrogenase (PGDH). Thus, luteolysis is initiated at days 17-20 of the normal bovine estrous cycle due to uterine secretion of PGF pulses. Some PGF arrives at the ovary, primarily through local pathways, and activates important molecular and cellular pathways that ultimately lead to CL regression (Davis and Rueda, 2002; Atli *et al.*, 2012; Maalouf *et al.*, 2014; *Ochoa et al., 201*8; Pate and Hughes, 2018).

Similar vascular anastomoses experiments demonstrated that pregnancy also maintains the CL through local pathways involving transport from uterine vein to ovarian artery. For example, transfer of embryos into a surgically-isolated uterine horn resulted in CL regression if the embryo was transferred contralateral to CL, but CL was maintained if embryo was transferred ipsilateral to the pregnancy in cows (Del Campo *et al.*, 1977) or ewes (Moor, 1968). In surgically-isolated horns, anastomoses of uterine veins from gravid to non- gravid side resulted in maintenance of CL on non-gravid side in both ewes (Mapletoft *et al.*, 1975) and cows (Del Campo *et al.*, 1980). This demonstrated that the pregnancy signal was local and not systemic and was carried in the local uterine vein. Other experiments were done with anastomoses of the ovarian artery from gravid to non-gravid side resulting in CL maintenance on non- gravid side (Mapletoft and Ginther, 1975; *Mapletoft et al., 1976b* ) demonstrating that the pregnancy signal passed from uterine vein to ovarian artery, only on the same side as the pregnancy (Fig. 1).

### Local pathways for CL maintenance during second month of pregnancy

Previous researchers stated *(Bridges et al.,* 2000), “a local relationship between the ovary bearing the CL and the embryo/fetoplacental unit still exists during the 2nd month of pregnancy” and we support this idea. The primary evidence that there is a local relationship comes from studies that have induced accessory CL on the contralateral or ipsilateral ovary during pregnancy. In one study (Lulai *et al.*, 1994), ten pregnant beef heifers were treated on days 30-35 of pregnancy with progestin implants, to maintain pregnancy, the original CL was regressed using cloprostenol, and heifers were given two treatments with LH to induce accessory CL. All heifers ovulated and had an accessory CL; however, half of these heifers regressed this CL by 15-17 days after LH treatment (day 45-50 of pregnancy). Of particular interest, the accessory CL was contralateral to the pregnancy in all five of the heifers that regressed, whereas, four of five of the heifers that maintained their CL had an accessory CL that was ipsilateral to the pregnancy (Lulai *et al.*, 1994). Similarly, induction of an accessory CL on days 29 to 59 of pregnancy was sufficient to maintain the pregnancy in 20 of 27 cows that had accessory CL that were ipsilateral to the pregnancy but maintained pregnancy in 0 of 5 cows that had accessory CL contralateral to the pregnancy (Bridges *et al.*, 2000). Thus, an induced CL contralateral to the pregnancy is not maintained and does not maintain the pregnancy during the second month.

Our research group induced accessory CL in lactating cows by treatment with GnRH on day 5 after AI (Baez *et al.,* 2017). In this model, 65.4% of cows (234/358) ovulated to the GnRH and formed an accessory CL. In pregnant cows, the accessory CL ipsilateral to the pregnancy rarely regressed (8/67), however when the accessory CL was contralateral to the pregnancy, most CL regressed (66.2%; 86/130) even though the pregnancy and original ipsilateral CL were maintained. Timing of contralateral CL regression is particularly relevant with only 25.6% (22/86) of contralateral CL regressions occurring during the first month and 74.4% (64/86) happening during the second month of pregnancy (P < 0.0001; Baez *et al.*, 2017). One interesting question is why some accessory CL regression happened during the normal time of maternal recognition of pregnancy (i.e. first month of pregnancy) while most happened during the second month. By day 18-20 of pregnancy, the whole gravid horn is filled by the elongating embryo and it extends into the contralateral horn during the next few days of pregnancy (Chang, 1952). It seems likely that the elongating embryo and IFNT will readily migrate into the contralateral horn in cows with a normal size uterus, such as heifers and primiparous cows. Interestingly, cows that had accessory CL regression during the first month of pregnancy were primarily multiparous cows. Multiparous cows have a much larger uterus than primiparous cows and fertility decreases as uterine size increases (Baez *et al.*, 2016; Young *et al.,* 2017). The coverage of the contralateral horn by embryonic membranes might be affected by uterine size and this may determine if early contralateral CL regression will occur. Nevertheless, most cows had contralateral, accessory CL regression during the second month of pregnancy (Baez *et al.*, 2017). We also induced accessory CL in heifers and, although no ipsilateral accessory CL regressed, almost all contralateral accessory CL regressed and this occurred primarily during the second month of pregnancy (Baez, Garcia-Guerra, Wiltbank, 2018, University of Wisconsin- Madison : unpublished). Thus, there is a second distinct period of either luteolysis or CL protection happening between days 30-60 of pregnancy, regulated by local, but likely distinct, pathways from those that have been described during the first month of pregnancy.

## Role of embryonic production of IFNT in maintenance of CL

In cyclic ewes, intrauterine infusion of homogenates or secreted proteins from day 14-15 embryos extended CL lifespan, while, homogenates of day 21-25 embryos did not alter CL lifespan (Rowson and Moor, 1967; Godkin *et al.*, 1984b), demonstrating the limited interval during pregnancy when the CL- maintaining signal is secreted by the conceptus. The active principal in the homogenates was heat and protease-labile, and had properties consistent with a low molecular weight protein (Rowson and Moor, 1967; Martal *et al.*, 1979; Godkin *et al.*, 1982). Later studies showed that a single protein, initially called ovine or bovine trophoblast protein-1 but later IFNT, was solely responsible for maintenance of the CL during pregnancy in ruminants (Godkin *et al.*, 1984a, 1997; *Thatcher et al., 1984* ).

Thus, during the critical period, days 17 to 25 in cattle or day 13-21 in sheep, the embryo is dramatically elongating and secreting IFNT, the definitive signal for CL maintenance during early pregnancy (Roberts, 1996; Bazer *et al*., 1997). In the uterus, INFT acts in a paracrine manner to prevent expression of estrogen receptor alpha and oxytocin receptor in luminal epithelial cells of the endometrium and superficial glandular epithelium, thereby altering response to oxytocin and release of luteolytic pulses of PGF (Spencer *et al.*, 2007). Interferon-tau also stimulates expression of specific genes, termed interferon-stimulated genes (ISG) in the uterus (Johnson *et al.*, 1999; Hansen *et al.*, 2013) although their role in maintenance of the CL has not been clearly defined.

In sheep, IFNT is not secreted by morula stage embryo but expression is detected in day 8 blastocysts with dramatic increases as the embryo begins to expand and elongate (Farin *et al.*, 1990; Ealy *et al.*, 2001). Substantial IFNT protein secretion is observed on day 12-13 of pregnancy, with 27-fold increases between days 13-17, with a subsequent decrease of 50% by day 21 (Hansen *et al.*, 1985). The mRNA for IFNT is maximal on day 14 of ovine pregnancy and is related to expression of two genes, IFNT1 and IFNTc1 (*Kim et al., 2018* ). The transcription regulatory pathways for induction of IFNT in the elongating embryo are well- described with ETS2 as the master regulator, combined with activation of a nearby AP-1 site and a DLX3 binding site (Ezashi and Imakawa, 2017). Maximal IFNT protein secretion occurs 2-3 days later with subsequent decreases in both IFNT mRNA and protein expression (Nojima *et al.*, 2004). There is disappearance of IFNT mRNA and protein by days 20-23 of ovine pregnancy (Godkin *et al.*, 1982; Guillomot *et al.,* 1990). One of the more interesting recent findings is the clear demonstrations that IFNT escapes the uterus and induces ISG expression in peripheral tissues such as the CL (Oliveira *et al.*, 2008; Bott *et al.*, 2010) and peripheral blood cells (Gifford *et al.*, 2007; *Shirasuna et al., 2012* ). Elegant experiments were done in which IFNT was delivered into the uterine vein (20 µg/day) or jugular vein (200 µg/day). These treatments increased expression of a number of genes in the CL including ISGs and cell survival genes (BCL2L1, Bcl-xL, AKT, and XIAP) and decreased PGF receptor expression (Antoniazzi *et al.*, 2013). In addition, ewes were challenged with a single injection of PGF (4 mg/58 kg body weight, i.m.). Ewes receiving infusions of BSA, as a control, had a decrease in circulating P4 to about 30% of control ewes that were not treated with PGF; however, IFNT-treated ewes that were also treated with PGF had only a small decrease in circulating P4 and a return to control values by 48 h after PGF treatment (Antoniazzi *et al.*, 2013). Thus, IFNT has clear actions within the uterus to decrease uterine PGF secretion. Embryonic IFNT also escapes the uterine lumen, acting directly on the CL, potentially to directly suppress the luteolytic actions of PGF on the CL. The actions of IFNT directly on the CL could be independent or synergistic with the actions of IFNT in the uterus.

Studies indicate decreased IFNT production after 3 weeks of pregnancy in sheep and an absence of IFNT in the second month of pregnancy in both cattle and sheep (Ealy and Yang, 2009; Ezashi and Imakawa, 2017). This sudden drop in IFNT mRNA occurs concurrently with attachment of the trophectoderm to the uterus (Guillomot *et al.*, 1990). The mechanisms are temporally linked to placental development and uterine attachment and these processes may mediate the reduction in IFNT expression. It is known there is increased DNA methylation in the 5’ flanking region of the IFNT gene in CpG islands that are proximal to the IFNT-010 gene (Nojima *et al.*, 2004). Culture of ovine embryos with a DNA methylation inhibitor results in no change in IFNT secretion in day 14 embryos but increased IFNT mRNA in day 17 embryos (*Nojima et al., 2004* ). Conversely, acetylation of histone H3 was observed in the IFNT gene near the CDX2-binding site on days 14-16 when IFNT expression was elevated but acetylation dramatically declined as the embryo attached to the uterine wall and IFNT declined (Sakurai *et al.*, 2010). Finally, the transcription factor EOMES has also been implicated in IFNT gene suppression since it increases near time of IFNT gene silencing and binds the coactivator CREBBP, thereby disrupting activation of the AP1 binding site in the 5’ flanking region of IFNT genes (Sakurai *et al.*, 2013). Thus, increased EOMES protein combined with declining histone acetylation and increased DNA methylation of the upstream region of the IFNT gene are linked to silencing of IFNT gene transcription as the embryo develops beyond day 17 of ovine pregnancy (Ezashi and Imakawa, 2017).

In cattle, there are some results that corroborate the timing of IFNT expression and subsequent silencing during early pregnancy. Expression of ISGs has been used as an indirect pregnancy marker at 16 to 22 days after AI (Romero *et al.,* 2015; Wijma *et al.*, 2016; Wiltbank *et al.*, 2016a) with a subsequent decrease near the expected time of conceptus attachment to the uterine lining. Kizaki *et al*. (2013) collected bovine blood samples from days 0 to 28 after AI and evaluated expression of ISGs using qPCR and microarray of mRNA from peripheral blood leukocytes. As pregnancy progressed (between days 21-28), peripheral blood leukocytes had a decrease in expression of ISGs, such as ISG15, MX1, MX2, and OAS1. Moreover, in another study (Pugliesi *et al.*, 2014), the abundance of ISG mRNA in maternal peripheral blood mononuclear cells (PBMC) peaked on day 20 and then had a sharp decrease, especially after day 30 post-AI in pregnant cows. Thus, in both cattle and sheep, most of the evidence indicates that IFNT is high during the maternal recognition of pregnancy period but then decreases to minimal secretion prior to the second month of pregnancy.

## Alterations in oxytocin-induced PGF pulses during pregnancy

### Patterns of PGFM during first month of pregnancy

The PGF patterns in ruminants are the result of communication between multiple organs using multiple hormones. The uterus is the central player and the source of PGF secretion. However, the posterior pituitary that releases pulses of oxytocin throughout the day is the source of the pulsatile pattern. Levels of oxytocin receptor in the uterus vary throughout the cycle, decreasing mid cycle, and increasing at day 17 through 21 in non-pregnant cows, with temporal association to uterine PGF secretion (Fuchs *et al.*, 1990; Mann and Lamming, 2006). The cascade of events that ultimately leads to expression of uterine oxytocin receptors involves an initial down-regulation of endometrial P4 receptors after continuous exposure to P4 during the luteal phase (Wathes *et al.*, 1996; Spencer *et al.*, 2004). This produces an up-regulation of uterine estrogen responsiveness, which, after activation of the estrogen receptor (ESR1) by circulating estradiol (E2) from a developing follicle, generates increased expression of oxytocin receptors (Ivell *et al.*, 2000; Mann *et al.*, 2001; Kieborz-Loos *et al.*, 2003; Fleming *et al.*, 2006). The key role of follicular E2 is highlighted by the delay in luteolysis when follicular E2 is reduced by either follicular aspiration (Araujo *et al.*, 2009) or inhibition of follicle growth with steroid-stripped follicular fluid (Salfen *et al.*, 1996). The essential role of oxytocin is evidenced by inhibition of PGF secretion and delay in luteolysis by treatment with an oxytocin receptor antagonist (Mann *et al.*, 2003). Binding of oxytocin to its receptor in the uterus stimulates PGF production via stimulation of both Phospholipase A2 and C (Burns *et al.*, 1997a), releasing arachidonic acid that is subsequently converted to PGH2 by prostaglandin H synthase-2, which is also induced by oxytocin (Burns *et al.,* 1997b).

Pulsatile secretion patterns of PGF have generally been evaluated by serial evaluations of the PGF metabolite (PGFM) throughout the process of CL regression (Wolfenson *et al.*, 1985; Silvia and Raw, 1993; Shirasuna *et al.*, 2004). While the pulsatile nature of these patterns is a consistent finding in all of these studies, the number of pulses and day when the pulses begin varies between animals. While CL regression can be triggered with a single large dose of exogenous PGF, the pulsatile nature of PGF secretion in the physiologic state has been shown to be required for regression when using smaller doses of PGF which more closely reflect the physiologic amounts present during regression (Ginther *et al*., 2009).

The PGFM concentrations have also been evaluated in early pregnant ruminants, near the time when luteolysis would normally occur in non-pregnant animals but fewer studies and inconsistent results have been reported. In one study (Lewis *et al.*, 1977), there were no detectable differences in mean concentrations of PGFM in blood from the jugular vein in pregnant vs non-pregnant ewes from day 11-16. However, in another study (Zarco *et al.*, 1988) mean concentrations of PGFM in pregnant ewes were greater than non- pregnant ewes on days 13-17. This study also provided some evidence for PGFM pulses in pregnant ewes, though they were of lower amplitude and less frequent than pulses documented in non-pregnant ewes (*Zarco et al., 1988* ). In the cow, one study (Kindahl *et al.*, 1976), failed to observe increases in PGFM concentrations or pulses of PGFM in pregnant animals during the time when luteolysis would be occurring in non-pregnant animals. However a recent study (Pinaffi *et al.*, 2018), described statistically-detectable PGFM pulses in pregnant heifers during this same time period, although the amplitude of PGFM pulses was much lower in pregnant than non-pregnant cows. There have been fewer studies done in goats, however, no pulses of PGFM were detected in pregnant does (Fredriksson *et al.*, 1984).

**Figure 2:**
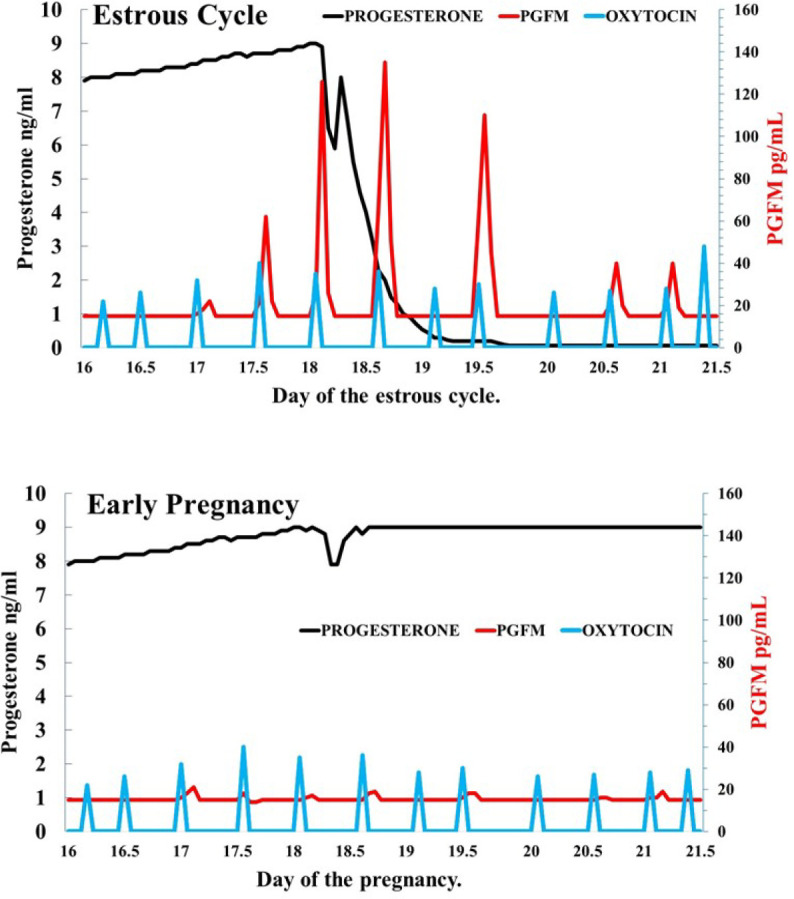
Simplified model for the patterns of PGFM, progesterone, and oxytocin in cattle during the late estrous cycle when luteolysis is occurring or during the same time in early pregnancy.

While not directly measuring PGFM production, there is documentation that from days 17-21 of bovine pregnancy there are decreased endometrial oxytocin receptors (Fuchs *et al.*, 1990). In ruminants, IFNT during the first month of pregnancy decreases expression of oxytocin receptors in endometrium and this mechanism appears to underlie the decrease in uterine oxytocin receptors during early pregnancy and inhibition of oxytocin-induced PGF secretion from the uterus (Jenner *et al.*, 1991; Asselin *et al.*, 1997; Dorniak *et al.*, 2011; Antoniazzi *et al.*, 2013; Romero *et al.*, 2015). It appears that IFNT does not prevent oxytocin receptor gene transcription directly, however, IFNT inhibits ESR1 transcription *in vitro*, preventing estrogen from being able to upregulate oxytocin receptor, and thereby blocking luteolytic amounts of PGF from being synthesized in pregnant animals (Spencer and Bazer, 1996; Spencer *et al.*, 1996; Fleming *et al.*, 2006). Consequently, there is decreased PGF in the uterine vein and ovarian artery and decreased pulsatile secretion of PGF by the uterus during early pregnancy (*Arosh et al., 2004;*Dorniak *et al.*, 2011). Thus, in Fig. 2 is shown the postulated difference in PGFM secretion patterns during the period encompassing the normal time of luteolysis in non-pregnant cows or a similar time of early pregnancy (Fig. 2).

### Patterns of PGFM during second month of pregnancy

Very little characterization of PGF patterns has occurred in ruminants during the second month of pregnancy. There is evidence that signaling pathways involved in PGF production become reestablished in the uterus and are present during month two of pregnancy. This idea is primarily supported by studies that used oxytocin challenges. During early pregnancy, oxytocin challenge on day 18 increased PGFM at 60 min in non- pregnant but not in pregnant heifers (Parkinson *et al.*, 1990; Robinson *et al.*, 1999). One study evaluated if this oxytocin response returns after the first month of pregnancy. Fuchs *et al.* challenged cows at known stages of gestation (50, 150, 250, or 280 days) with 10 or 100 IU oxytocin, and measured circulating oxytocin and PGFM during the next 3 h (Fuchs *et al.*, 1996). Circulating oxytocin concentrations in plasma were not affected by gestation length, however PGFM increased after oxytocin challenge as dose and time of pregnancy increased. For example, at day 280 there was 7-fold greater increase in circulating PGFM than on day 50, although on day 50 there was a clear increase in PGFM after 100 IU of oxytocin. In addition to the oxytocin challenge, presence of intercaruncular endometrial oxytocin receptors were found at day 50 of pregnancy, suggesting the uterus is capable of responding to oxytocin stimulation with PGF production during the second month of pregnancy (Fuchs *et al.*, 1996).

Another study evaluated this topic by maintaining pregnancies for the first month of pregnancy using exogenous progestins and by inducing an accessory CL during the second month of pregnancy. Cows on days 31-35 of pregnancy had a tendency to maintain the pregnancy at a greater rate if they produced more PGFM, as measured in blood from the posterior vena cava, and these cows also had greater circulating P4 (Bridges *et al.*, 2000). This study also reported constant secretion of PGF, rather than secretory episodes in samples taken hourly during a 10 h period (Bridges *et al.*, 2000). However, it is possible that the multiple treatments done in this study, such as regression of the original CL and formation of an accessory CL, may have had some residual effects on the PGF secretion patterns.

Recent data from our group (Drum, Wiltbank, Sartori, 2018; University of Wisconsin-Madison; unpublished) showed that the upregulation of oxytocin receptors occurs even earlier during pregnancy. By day 25 after AI in pregnant lactating dairy cows, there was 3-fold increase in circulating PGFM concentrations 30 min after treatment with 50 IU oxytocin i.m. but no detectable PGFM increase at day 18 in pregnant cows. The response to oxytocin challenge was increased as pregnancy progressed until day 53-60. Moreover, no cow aborted after oxytocin treatment, despite substantial increases in circulating PGFM. These intriguing data argue that in the absence of IFNT after day 18 to 21 of pregnancy, endometrial oxytocin receptors increase, allowing for PGF release in response to oxytocin.

**Figure 3:**
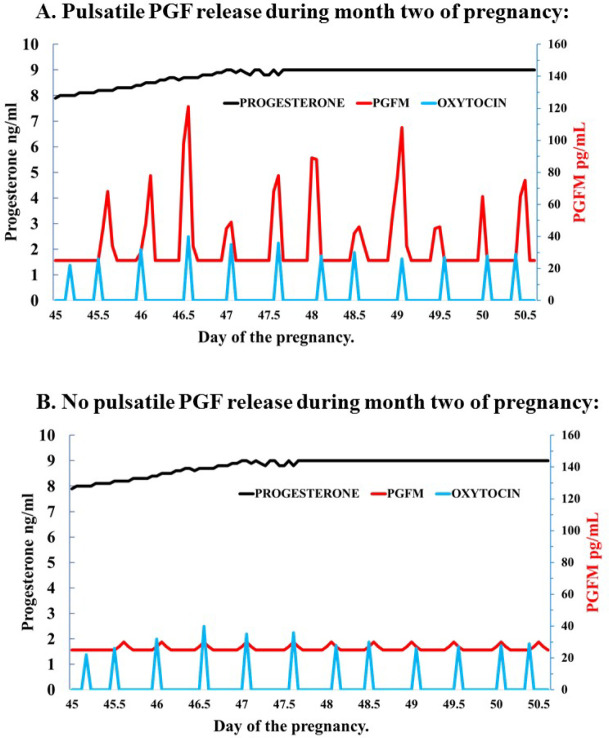
Speculation on two different models for the patterns of PGFM, progesterone, and oxytocin in cattle during the second month of pregnancy.

Thus, there are two distinct models for circulating PGFM during second month of pregnancy: 1) PGFM pulses are present during the second month of pregnancy (Fig. 3A), consistent with the presence of uterine oxytocin receptors and oxytocin-induced PGFM secretion in pregnant cows after day 25 (Fuchs *et al.*, 1996) or 2) no pulses of PGF during the second month of pregnancy, consistent with (Bridges *et al.*, 2000), in spite of pulsatile oxytocin secretion (Fig. 3B). Further research will be necessary to determine which model is correct for month two of pregnancy.

## Protection from PGF action during first and second month of pregnancy

A third mechanism that may protect the CL of pregnancy is protection from luteolytic effects of the uterine luteolysin, PGF. This idea was based on the results of experiments primarily done with pregnant and non-pregnant ewes starting in the mid-1970s by three research groups. At University of Wisconsin, the research group of OJ Ginther showed that a pregnancy substance was present in either the utero-ovarian vein (UOV) or ovarian artery (OA) on the same side as the early pregnancy (before day 20) but not in the contralateral vessels and this substance had a luteotropic effect or, more accurately in our opinion, an antiluteolytic effect (Mapletoft *et al.*, 1975,1976b). In these intricate experiments it was clearly shown that the pregnant uterus produces a substance that is transported in the UOV and subsequently in the OA and that this substance protects the CL from the luteolytic actions of endogenous or exogenous PGF.

At University of West Virginia two studies were done using a model in which a low dose of PGF was injected into the largest follicle near the CL in pregnant or non-pregnant ewes (Inskeep *et al.*, 1975; Pratt *et al.*, 1977a). A dose of 200 µg of PGF, given on day 12 of estrous cycle or pregnancy, induced luteolysis in 11/14 non-bred ewes and in 8/9 mated ewes that did not have an embryo, however, it induced luteolysis in only 3/8 mated ewes with an embryo (2 ewes with CL regression had a very small embryo; Inskeep *et al.*, 1975). In a second experiment (Pratt *et al.*, 1977a), ewes on day 13 of the estrous cycle or pregnancy received 270 µg of PGF-Tham salt or saline in the ipsilateral dominant follicle and luteolysis was induced in 8/8 non- bred ewes and in 5/5 mated ewes without an embryo but in none of the 6 mated ewes that had an embryo.

Finally, a series of experiments were done at Colorado State University, by Bill Silvia and Gordon Niswender to evaluate whether the CL of early pregnancy was resistant to the effects of exogenous PGF. First, various doses of PGF were tested and they found that a dose of 4 mg PGF/58 kg of body weight i.m., given on day 13, caused CL regression in 5/8 non- pregnant ewes but in 0/9 pregnant ewes (Silvia and Niswender, 1984). In a subsequent experiment, they used this same PGF dose in pregnant and non-pregnant ewes on day 10 or 13 (Silvia and Niswender, 1986) and found that PGF decreased P4 in both pregnant and non- pregnant on day 10 but only regressed the CL in non- pregnant ewes on day 13. In the final experiment, ewes were treated on various days of pregnancy with the same PGF dose (Silvia and Niswender, 1986). They found no effect of PGF at 36 h after treatment on P4 in ewes on days 13 or 16 of pregnancy but major decreases in P4 on days 10, 26, and 30 of pregnancy. Day 19 and 22 of pregnancy had an intermediate response to this low dose of PGF. Thus, resistance to PGF is only present during a limited period of pregnancy, specifically when IFNT is being secreted by the embryo, and prior to that time or after that period there is no detectable resistance to PGF action caused by pregnancy. In this regard, a similar model was utilized to evaluate PGF resistance during endocrine delivery of IFNT in non-pregnant ewes on day 10 of the estrous cycle (Antoniazzi *et al.*, 2013). Similar to early pregnancy, endocrine delivery of IFNT inhibited the action of PGF on circulating P4, suggesting that actions of IFNT cause the PGF resistance of early pregnancy.

There is also evidence from field studies that there is PGF resistance during early pregnancy in ewes. In one study, with 270 presumably pregnant ewes, 90 ewes were treated with PGF (125 µg cloprostenol) at 22-23 days post-service. The remaining 180 bred ewes were used as lambing controls (Reid and Crothers, 1980). Almost all controls (98.9%) subsequently lambed, whereas, only 36.7% of the PGF-treated ewes lambed. Thus, PGF was effective in aborting most pregnant ewes (63.3%) but about one-third of pregnant ewes displayed a resistance to PGF treatment at this stage of pregnancy. Another research group (Nancarrow *et al.*, 1982), treated ewes on day 21 of pregnancy with 100 µg of cloprostenol and found that 15/23 of these ewes maintained their pregnancy. Interestingly, the ewes that had PGF resistance (i.e. maintained their pregnancy) had significantly more CL (3.2) than ewes that had CL regression (1.8) suggesting that a greater mass of embryonic tissue provides greater PGF protection. The number of CL, alone, did not provide protection from PGF since 93.5% (43/46) of non- pregnant ewes had CL regression to this dose of PGF, and all non-pregnant ewes with more than one CL regressed their CL. Thus, the CL of early pregnancy is somewhat resistant to PGF action due to the action of a substance coming from the embryo.

A number of reports indicate that the locally- active, luteoprotective factor is likely to be PGE2 or PGE1 (PGE) secreted from the endometrium in response to pregnancy or IFNT (Huie *et al.,* 1981; Arosh *et al.*, 2004,^2016^; Weems *et al.*, 2011). One type of evidence is that during pregnancy, the bovine or ovine uterus produces much greater amounts of PGE than during a similar time period in non-pregnant animals (Danet-Desnoyers *et al.*, 1995; Arnold *et al.*, 2000; Lee *et al.*, 2012). In addition, PGE is also produced by ovine (Hyland *et al.,* 1982; *Charpigny et al., 1997* ) and bovine (Saint-Dizier *et al.,* 2011) embryos during early pregnancy. It has also been found that PGEs (Huie *et al.,* 1981; Lee *et al.,* 2012) can diffuse through the utero-ovarian plexus and provide local protective effects on the CL during establishment of pregnancy in sheep. Of particular importance, many studies have reported that treatment with PGE can inhibit the luteolytic actions of PGF in ovine and bovine CL (Henderson *et al.*, 1977; Pratt *et al.*, 1977b; *Huie et al., 1981;*Reynolds *et al.*, 1981). Recently we have found that pulses of extremely low doses of PGF, delivered into the uterus, can cause complete CL regression and expression of an intriguing cascade of gene expression that is likely to be critical for luteolysis (Atli *et al.*, 2012; Ochoa *et al.*, 2018). Of special importance, simultaneous intrauterine infusion of PGE with PGF completely blocked the actions of PGF on CL regression and PGF-induced luteal gene expression. These results indicate that either PGE blocks transport of PGF to the CL, the model that we favor, or PGE completely blocks PGF action at the CL level. Thus, the temporal secretion pattern of PGE during early pregnancy, the chemical nature of PGE that is consistent with local delivery of the antiluteolytic substance, and the biological actions of PGE to block PGF action are consistent with PGE being responsible for the observed resistance to PGF action during early pregnancy.

In contrast to early pregnancy, the CL during the second month of pregnancy does not seem to be resistant to PGF action. This is evidenced by the finding that the CL is resistant to PGF action during day 13-16 of pregnancy but loses this resistance by day 26 of pregnancy in sheep (Silvia and Niswender, 1986). Further, treatment with PGF (500 µg cloprostenol) during the second month of pregnancy (day 45-60) caused abortion in 97.9% of pregnant cows (47/48); although this same dose of PGF has not been evaluated in cows during early pregnancy (Thain, 1977).

**Figure 4:**
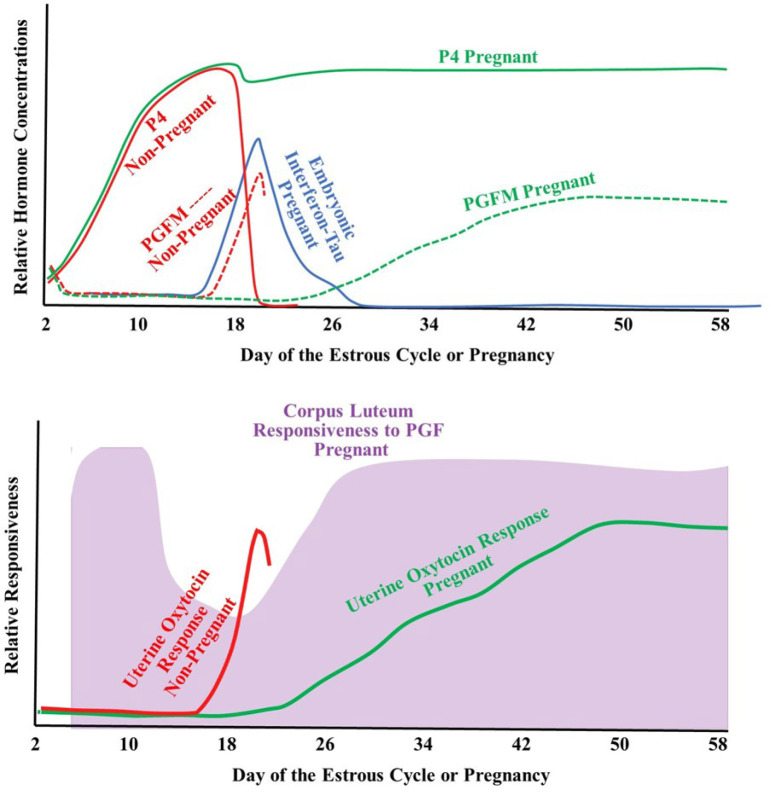
Model of relative changes in hormonal concentrations (upper graph) and relative changes in responsiveness (lower graph) of the CL to PGF (based on extrapolated data from sheep in Silvia and Niswender (1986) or the uterus to oxytocin in pregnant and non-pregnant cows.

Thus, there is substantial evidence that PGF resistance occurs during early pregnancy (IFNT period) but little evidence for PGF resistance during the second month of pregnancy (See representation in Fig. 4: CL sensitivity to PGF). Most of this evidence is from studies using pregnant ewes with a paucity of research evaluating whether PGF resistance occurs in cattle during early pregnancy. The mechanisms that underlie this PGF resistance has been investigated with no evidence for changes in PGF receptors (Wiepz *et al.*, 1992) but substantial evidence that there are decreases in PGF synthesis and increased PGF degradation (increased PGDH enzyme) in early pregnant ewes compared to ewes at similar stages of the estrous cycle (Silva *et al.*, 2000; Costine *et al.*, 2007; Lee *et al.*, 2012).

## Conclusions on maintenance of the CL during first or second month of pregnancy

**Figure 5:**
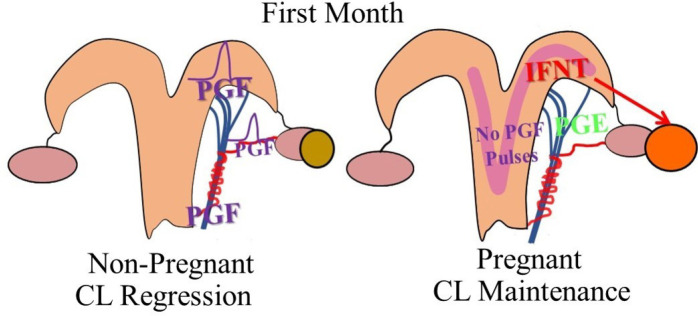
Physiological model depicting the three potential mechanism protecting the CL during the first month of pregnancy in ruminants. In non-pregnant ruminants, the uterus secretes prostaglandin F2α (PGF) which is secreted in discrete pulses and arrives at the corpus luteum (CL) via transport from the utero-ovarian vein to the ovarian artery. In the pregnant ruminant, the elongating embryo secretes interferon-tau (IFNT) which acts to maintain the CL by: 1) suppressing PGF pulses, 2) increasing prostaglandin E (PGE) production, 3) IFNT can escape from the uterus and act directly on the CL.

In Fig. 4 is shown a summary of results discussed in the previous sections and normalized to the events that occur during bovine pregnancy. During early pregnancy, circulating P4 is increasing in both pregnant and non-pregnant cows. Near day 18 of the estrous cycle, there is an increase in uterine responsiveness to oxytocin and a dramatic increase in PGFM pulses in non-pregnant cows that leads to rapid CL regression and initiation of a new estrous cycle. In pregnant cows during this same time period there is an increase in IFNT secretion by the early embryo which inhibits the induction of oxytocin receptor expression in the pregnant uterus and therefore uterine responsiveness to oxytocin is inhibited and oxytocin-induced PGFM pulses are inhibited. In addition, there is some evidence from early pregnant sheep that the CL becomes relatively unresponsive to PGF, perhaps due to PGE secretion from the pregnant uterus. Thus, the CL of early pregnancy is maintained by multiple mechanisms that are initiated by IFNT, seem to be primarily mediated by local pathways, and seem to involve decreased PGF secretion, perhaps decreased PGF transport, and clearly less PGF action at the CL. Figure 5 illustrates a physiological model for the three main mechanisms that are maintaining the CL during the classical maternal recognition of pregnancy that occurs in the first month of pregnancy.

However, the mechanisms that are protecting the CL of early pregnancy do not appear to persist into the second month of pregnancy. It appears that IFNT is no longer secreted by the embryo after the apposition and attachment process are initiated. Therefore, it appears that uterine responsiveness to oxytocin returns during the second month of pregnancy, basal PGF secretion increases and, perhaps, oxytocin-induced PGF pulses are reinitiated during the second month of pregnancy. In addition, CL responsiveness to PGF appears to return during the second month of pregnancy after the loss of IFNT. Thus, current evidence is consistent with the idea that none of the mechanisms that protect the CL during the first month of pregnancy are protecting the CL during the second month of pregnancy.

In our opinion, there are three logical explanations that could explain the maintenance of the CL during the second month of pregnancy. First, there is a luteoprotective substance that is secreted by the pregnancy during month two and it protects the CL either directly or by inhibiting arrival of active PGF at the CL. Since the evidence from contralateral CL regression indicates that luteal protection is locally mediated (ipsilateral CL protected, contralateral CL generally regresses), a compound like PGE would be logical. A second possibility is that PGF is secreted from the non-gravid horn in response to oxytocin but there is no PGF secretion from the gravid horn during the second month of pregnancy. This idea has not been tested and therefore remains a possibility.

**Figure 6:**
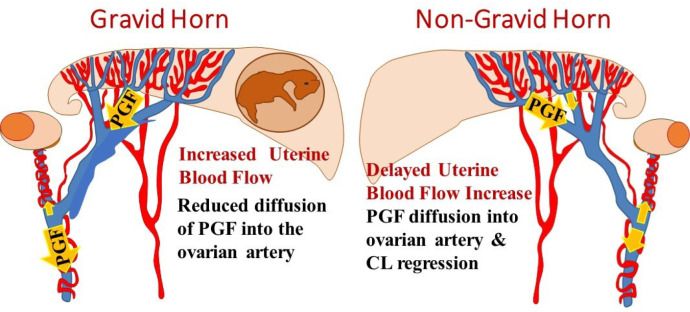
Current speculative physiological model depicting a mechanism that could be protecting the CL during the second month of pregnancy and possibly later pregnancy in ruminants. The gravid horn has an increased uterine blood flow during pregnancy and this high blood flow may inhibit transfer of uterine-secreted PGF to the ovarian artery. Therefore the CL would not regress because it would not be exposed to sufficient PGF, even during pulses of PGF, due to the reduction or loss of the local PGF transport system. In the contralateral horn, there is a delay in development of placentomes and the pregnancy-induced increase in uterine blood flow. Thus, contralateral CL because the local transport system for PGF will still be functional, allowing contralateral CL regression during the second month of pregnancy, as observed in previous studies.

The physiological model that we currently favor for protection of the CL during the second month of pregnancy is shown in Fig. 6. This model postulates that the increase in uterine blood flow that accompanies development of the placentomes also inhibits the local transport of PGF into the ovarian artery. This could be due to changes in the PGF transporter or may be related to decreased diffusion times or increased diffusion distances for PGF from the UOV to the OA. In the non- gravid horn, a reduced uterine blood flow, allows local transport of PGF which is increasing during the second month of pregnancy. This leads to regression of the CL when PGF pulses that reach the ovary are of sufficient magnitude and frequency. Obviously, future research is needed to definitively evaluate the many aspects of this intriguing but highly speculative physiological model.
